# Supplementary material for: Inversion and computational maturation of drug response using human stem cell derived cardiomyocytes in microphysiological systems
Source: Sci Rep. 2018 Dec 4;8:17626. doi: 10.1038/s41598-018-35858-7 (PMC6279833; doi:10.1038/s41598-018-35858-7)
Supplement: Supplementary file 1 — Supplementary Data [file 41598_2018_35858_MOESM1_ESM.pdf]

Supplementary information:
Inversion and computational maturation of drug response using human stem cell derived cardiomyocytes in microphysiological systems

Aslak Tveito[1], Karoline Horgmo Jæger[1], Nathaniel Huebsch[2], Berenice Charrez[2], Andrew G. Edwards[1,3], Samuel Wall[1], and Kevin E. Healy[2]

[1]Simula Research Laboratory, Norway
[2]Departments of Bioengineering, Material Science and Engineering, University of California, Berkeley, California, USA
[3]Department of Biosciences, University of Oslo, Norway

October 5, 2018

1 Modification of the action potential model

In the process of adjusting the Paci et al. [1] model to the data obtained from an MPS (microphysiological system, [2]), we have to run the model many thousand times with varying choices of parameters. One difficulty encountered in this process is drift of ion concentrations. This is a well-known problem of mathematical models of electrophysiology; see e.g. [3, 4, 5]. In Figure S1, we illustrate this problem for the original Paci et al. model. One approach to solve this problem is to decompose stimulus currents into ion concentrations and thereby retain conservation of the ion concentrations, see e.g. [3]. A problem with this approach is that drift is observed also when no stimulus is applied (see Figure S1). Another approach relies on the fact that some ion concentrations vary little and can therefore be kept constant. Here, we follow this latter approach and freeze the intracellular sodium concentration and the SR calcium concentration at their initial value. In Figure S2, we show the properties of this approximation. In the right panel, we note that the cytosolic calcium concentration no longer drifts even for very long simulations. In the left panel, we show that the effect of this approximation

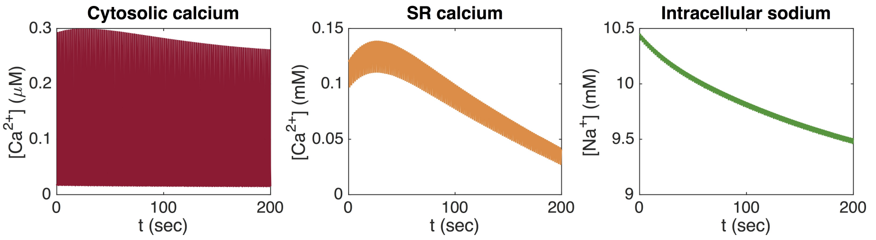

Figure S1: Example of drift of ionic concentrations in the Paci et al. model [1] with no stimulus current applied. First, we compute the steady state solution of the original Paci et al. model. Then, we reduce the I_{Kr} current by 50% and run a simulation of this adjusted model for 200 seconds (corresponding to approximately 120 AP cycles). The plots show how the cytosolic calcium concentration (left panel), the SR calcium concentration (center panel), and the intracellular sodium concentration (right panel) change with time during this long simulation.

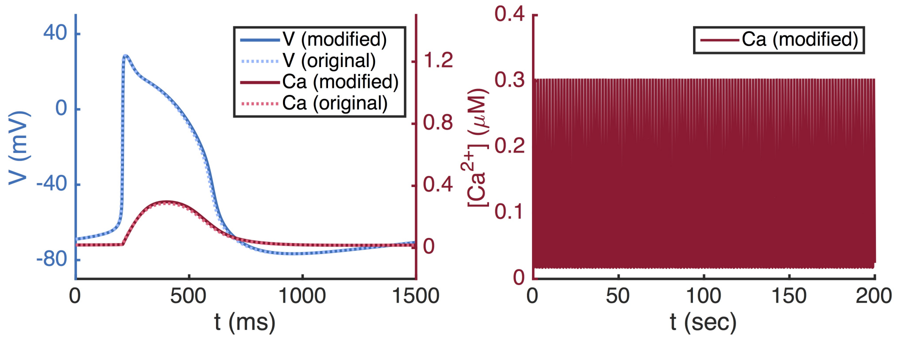

Figure S2: Effect of freezing the intracellular sodium concentration and the SR calcium concentration in the Paci et al. model [1]. Left panel: Comparison of the transmembrane potential and the cytosolic calcium concentration in the original Paci et al. model and the modified model with constant intracellular sodium and SR calcium concentrations. Right panel: Long-term effect on the cytosolic calcium concentration of reducing the I_{Kr} current by 50%. The corresponding effect in the original Paci et al. model is given in the left panel of Figure S1.

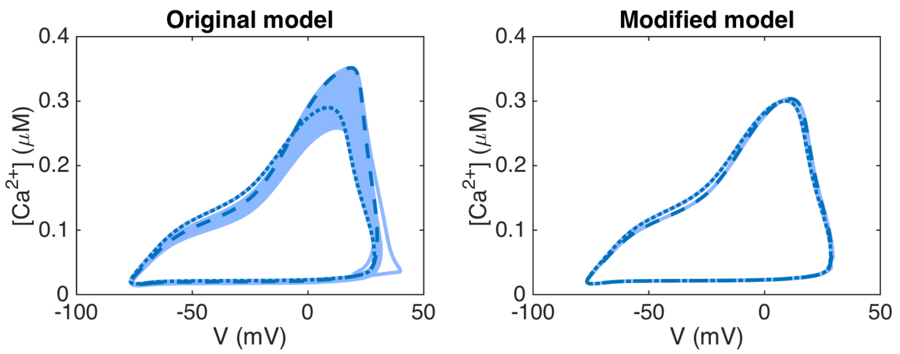

Figure S3: Convergence to steady state after scaling the I_{Kr} current down by 50%. We first run a single cycle of the original Paci et al. model, before running a simulation of the model with a reduced I_{Kr} for 5000 seconds (corresponding to approximately 3000 AP cycles). The cytosolic calcium concentration is plotted against the transmembrane potential of each cycle in light blue. The dotted line shows the cycle with the parameter values of the original Paci et al. model and the dashed line shows the new steady state solution obtained for a reduced I_{Kr}. Left panel: For the original Paci et al. model [1], a new steady state solution is not reached until after approximately 1000 AP cycles. Right panel: In the modified model with constant intracellular sodium and SR calcium concentrations, the solution does not change much after the first cycle with a reduced I_{Kr} current.

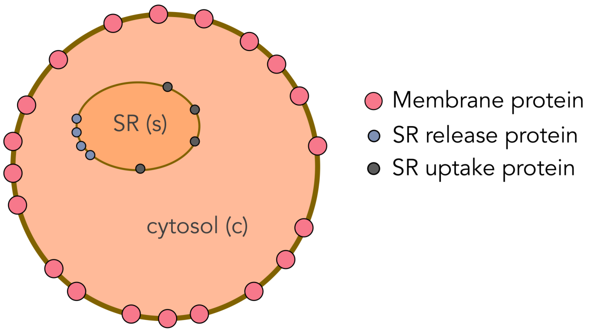

Figure S4: Illustration of the intracellular volume consisting of the cytosolic space (c) and the SR (s). The SR is equipped with specialized proteins for uptake and release of calcium.

on the transmembrane potential and the cytosolic calcium concentration is very small. With this approximation, convergence towards the steady state solution (a steady periodic solution) is rapid and the solutions appears to be stable. This is demonstrated in Figure S3 where convergence to steady state is illustrated. First, we compute the steady state solution of the modified model using the original parameters of the Paci et al. model. Then, we reduce I_{Kr} by 50% and note that the solution rapidly reaches equilibrium.

2 The maturation map of the Calcium dynamics

We consider how the Ca-dynamics change under maturation. As for the membrane ion channel case, we do this by illustrating the maturation process for a very simple model.

We consider an intracellular volume consisting of the cytosol (c) surrounding the sarcoplasmic reticulum (SR (s)); see Figure S4.

We let N_c denote the number of Ca^{2+}-ions in the cytosol and N_s denote the number of Ca^{2+}-ions in the sarcoplasmic reticulum; both given in mmol. The associated volumes are given by V_c and V_s, both given in L. Let $J_{c,s}$ and $J_{s,c}$ denote the flux (in mmol/ms) of Ca^{2+}-ions from the cytosol to the SR, and from the SR to the cytosol, - respectively. Conservation of Ca^{2+}-ions yields the model

$$\frac{dN_c}{dt} = J_{s,c} - J_{c,s}, \quad (1)$$

$$\frac{dN_s}{dt} = J_{c,s} - J_{s,c}. \quad (2)$$

The fluxes are models of proteins carrying ions from one volume to the other. Let

$g^0_{c,s}$ (in mmol/ms) be the flux representing one single protein transporting Ca^{2+}-ions from the cytosol to the SR. Similarly, $g^0_{s,c}$ (in mmol/ms) is the flux representing one single protein releasing Ca^{2+}-ions from the SR to the cytosol. The number of such proteins are given by $N_{c,s}$ and $N_{s,c}$. Then, the system (1) and (2) takes the form

$$\frac{dN_c}{dt} = N_{s,c}g^0_{s,c} - N_{c,s}g^0_{c,s}, \tag{3}$$

$$\frac{dN_s}{dt} = N_{c,s}g^0_{c,s} - N_{s,c}g^0_{s,c}. \tag{4}$$

By defining the fluxes (in mM/ms)

$$j_{c,s} = \frac{N_{c,s}g^0_{c,s}}{V_c}, \quad j_{s,c} = \frac{N_{s,c}g^0_{s,c}}{V_c}, \tag{5}$$

the system takes the form

$$\frac{dC_c}{dt} = j_{s,c} - j_{c,s}, \tag{6}$$

$$\frac{dC_s}{dt} = \frac{V_c}{V_s}(j_{c,s} - j_{s,c}), \tag{7}$$

where C_c and C_s are the concentrations (in mM) of Ca^{2+}-ions in the cytosol and SR, respectively;

$$C_c = \frac{N_c}{V_c}, \; C_s = \frac{N_s}{V_s}. \tag{8}$$

For maturation, we can now follow the same steps as for the membrane proteins. During maturation, the properties of the single proteins will remain constant, but the number of proteins and the volumes will increase. Therefore, we introduce constants $q_{V_c}, q_{V_s}, q_{N_{c,s}}$ and $q_{N_{s,c}}$ such that

$$V_c^M = q_{V_c}V_c^{IM}, \quad V_s^M = q_{V_s}V_s^{IM}, \tag{9}$$

$$N_{c,s}^M = q_{N_{c,s}}N_{c,s}^{IM}, \quad N_{s,c}^M = q_{N_{s,c}}N_{s,c}^{IM}. \tag{10}$$

With

$$j_{c,s}^{IM} = \frac{N_{c,s}^{IM}g^0_{c,s}}{V_c^{IM}}, \quad j_{s,c}^{IM} = \frac{N_{s,c}^{IM}g^0_{s,c}}{V_c^{IM}}, \tag{11}$$

we get

$$j_{c,s}^M = \frac{N_{c,s}^M g^0_{c,s}}{V_c^M} = \frac{q_{N_{c,s}}N_{c,s}^{IM}g^0_{c,s}}{q_{V_c}V_c^{IM}} = \frac{q_{N_{c,s}}}{q_{V_c}}j_{c,s}^{IM}, \tag{12}$$

$$j_{s,c}^M = \frac{N_{s,c}^M g^0_{s,c}}{V_c^M} = \frac{q_{N_{c,s}}N_{s,c}^{IM}g^0_{s,c}}{q_{V_c}V_c^{IM}} = \frac{q_{N_{s,c}}}{q_{V_c}}j_{s,c}^{IM}. \tag{13}$$

Consequently, we have the IM model

$$\frac{dC_c}{dt} = j_{s,c}^{IM} - j_{c,s}^{IM}, \tag{14}$$

$$\frac{dC_s}{dt} = \frac{V_c^{IM}}{V_s^{IM}}(j_{c,s}^{IM} - j_{s,c}^{IM}), \tag{15}$$

and the associated M model

$$\frac{dC_c}{dt} = \frac{q_{N_{s,c}}}{q_{V_c}} j_{s,c}^{IM} - \frac{q_{N_{c,s}}}{q_{V_c}} j_{c,s}^{IM}, \tag{16}$$

$$\frac{dC_s}{dt} = \frac{q_{V_c} V_c^{IM}}{q_{V_s} V_s^{IM}}(\frac{q_{N_{c,s}}}{q_{V_c}} j_{c,s}^{IM} - \frac{q_{N_{s,c}}}{q_{V_c}} j_{s,c}^{IM}). \tag{17}$$

Again, we observe that the M model is obtained simply by multiplication by a set of maturation factors.

3 Technical specifications of the model formulation and inversion procedure

In this section, technical specifications regarding the model formulation used in the simulations and the inversion procedure will be provided.

3.1 Intracellular concentrations

In almost all of our computations, we use the modified version of the Paci et al. model described above with fixed intracellular sodium and SR calcium concentrations. The only exception is that we also run some simulations of ten Tusscher et al. model [6] in Figure 9 of the paper. In these simulations, the intracellular potassium, sodium and SR calcium concentrations are also fixed at constant values. The intracellular concentrations used in the IM and M formulations of the modified Paci et al. model and the similarly modified ten Tusscher et al. model are given in Table S1.

3.2 Numerical stimulation protocol

In all simulations, the cells are stimulated every 1000 ms by a 5 ms long stimulus current of 8 μA/μF. The simulations are run for five AP cycles before recording the action potential and calcium transient for each new parameter combination.

	$[Na^+]_i$ (mM)	$[K^+]_i$ (mM)	$[Ca^{2+}]_{SR}$ (mM)
Paci IM	10.45	150.00	0.12
Paci M	10.45	150.00	0.55
ten Tusscher IM	11.37	138.20	0.12
ten Tusscher M	11.37	138.20	0.53

Table S1: Intracellular concentrations used in the IM and M versions of the modified Paci et al. [1] and ten Tusscher et al. [6] models with fixed intracellular sodium, intracellular potassium and SR calcium concentrations.

3.3 Technical specifications for the drug inversions

When the inversion procedure is used to fit simulated or measured drug and control data, we only consider adjustments of the q_{Na}, q_{CaL}, q_{Kr} and q_{K1} factors, unless otherwise specified. Note, however, that for the inversion of the Verapamil data in Figures 6 and 7 of the paper, the I_{Na} current was reduced by 50%, the I_{NaK} current was reduced by 60%, the I_{CaL} current was increased by 60%, and the I_{up} and I_{rel} fluxes were increased by 30% before running the inversion of the q_{Na}, q_{CaL}, q_{Kr} and q_{K1} factors, in order to make the base model used in the inversion more similar to the control data.

3.4 Technical specifications for the construction of the maturation map

In the construction of the maturation maps demonstrated in Figure 9 of the paper, we use the inversion procedure to fit an immature model (Paci et al. [1]) to a mature model (ten Tusscher et al. [6]) and to fit the mature model to the immature model. In these inversions, we consider adjustments of the q_{Na}, q_{CaL}, q_{to}, q_{Ks}, q_{Kr}, q_{K1}, q_{NaCa}, q_{NaK}, q_{pCa}, q_{f}, q_{bNa}, q_{bCa}, q_{leak}, q_{up}, and q_{rel} factors (in addition to the q_{pK}-factor for the ten Tusscher et al. model). Note that the I_{f} current is added to the ten Tusscher et al. model in these simulations using the same formulation as in the default Paci et al. model, but with a conductance reduced by a factor of 10 for the mature ten Tusscher model, i.e. $g_{\mathrm{f}} = 0.003$ mS/μF.

Because of the large number of free parameters, we conducted a more detailed inversion procedure in this case with twelve iterations and 15000 randomly chosen adjustment factors in each iteration. In addition, we included some additional terms in the cost function containing information that is not available from the optical measurements, but may be obtained from the mathematical models. More

specifically, we used a cost function of the form

$$H(\lambda) = \left(\sum_{j=1}^{20} H_j(\lambda) \right)^{1/2}, \qquad (18)$$

where $H_1 - H_8$ are the same as in the remaining applications of the inversion procedure, that is

$$H_1 = \frac{|\int_{t_0(\lambda)}^{t_1(\lambda)} v(\lambda)dt - \int_{t_0^*}^{t_1^*} v^* dt|}{|\int_{t_0^*}^{t_1^*} v^* dt|}, \qquad H_2 = \frac{|\text{APD}_{V,30}(\lambda) - \text{APD}^*_{V,30}|}{|\text{APD}^*_{V,30}|},$$

$$H_3 = \frac{|\text{APD}_{V,50}(\lambda) - \text{APD}^*_{V,50}|}{|\text{APD}^*_{V,50}|}, \qquad H_4 = \frac{|\text{APD}_{V,80}(\lambda) - \text{APD}^*_{V,80}|}{|\text{APD}^*_{V,80}|},$$

$$H_5 = \frac{|(\frac{dc}{dt})_{\max}(\lambda) - (\frac{dc}{dt})^*_{\max}|}{|(\frac{dc}{dt})^*_{\max}|}, \qquad H_6 = \frac{|\text{APD}_{\text{Ca},30}(\lambda) - \text{APD}^*_{\text{Ca},30}|}{|\text{APD}^*_{\text{Ca},30}|},$$

$$H_7 = \frac{|\text{APD}_{\text{Ca},50}(\lambda) - \text{APD}^*_{\text{Ca},50}|}{|\text{APD}^*_{\text{Ca},50}|}, \qquad H_8 = \frac{|\text{APD}_{\text{Ca},80}(\lambda) - \text{APD}^*_{\text{Ca},80}|}{|\text{APD}^*_{\text{Ca},80}|},$$

where the star $*$ is used to denote the simulated data to which we are trying to adjust the model. Furthermore, $\text{APD}_{V,30}$ is defined as the length (in ms) of the time from the value of the membrane potential, in the upstroke, is 30% below its maximum value (t_0) until it again is repolarized to 30% of its maximum value (t_1). In H_1, we compute the integral of the membrane potential with respect to time t from $t = t_0$ to $t = t_1$. The values $\text{APD}_{V,50}$ and $\text{APD}_{V,80}$ are defined similarly to $\text{APD}_{V,30}$, and the terms $\text{APD}_{\text{Ca},30}$, $\text{APD}_{\text{Ca},50}$ and $\text{APD}_{\text{Ca},80}$ represent the corresponding transient durations for the calcium concentration. Moreover, in H_5, $(\frac{dc}{dt})_{\max}$ is the maximal upstroke velocity of the calcium concentration.

The additional terms for the construction of the maturation map are given by

$$H_9 = \frac{|v(\lambda)_{\max} - v^*_{\max}|}{|v^*_{\max}|}, \qquad H_{10} = \frac{|v(\lambda)_{\text{rest}} - v^*_{\text{rest}}|}{|v^*_{\text{rest}}|},$$

$$H_{11} = \frac{|c(\lambda)_{\max} - c^*_{\max}|}{|c^*_{\max}|}, \qquad H_{12} = \frac{|c(\lambda)_{\text{rest}} - c^*_{\text{rest}}|}{|c^*_{\text{rest}}|},$$

$$H_{13} = \frac{||I_{\text{Na}}(\lambda) - I^*_{\text{Na}}||_2}{||I^*_{\text{Na}}||_2}, \qquad H_{14} = \frac{||I_{\text{Na}}(\lambda) - I^*_{\text{Na}}||_\infty}{||I^*_{\text{Na}}||_\infty},$$

$$H_{15} = \frac{||I_{\text{CaL}}(\lambda) - I^*_{\text{CaL}}||_2}{||I^*_{\text{CaL}}||_2}, \qquad H_{16} = \frac{||I_{\text{CaL}}(\lambda) - I^*_{\text{CaL}}||_\infty}{||I^*_{\text{CaL}}||_\infty},$$

$$H_{17} = \frac{||I_{\text{Kr}}(\lambda) - I^*_{\text{Kr}}||_2}{||I^*_{\text{Kr}}||_2}, \qquad H_{18} = \frac{||I_{\text{Kr}}(\lambda) - I^*_{\text{Kr}}||_\infty}{||I^*_{\text{Kr}}||_\infty},$$

$$H_{19} = \frac{\|I_{\mathrm{K1}}(\lambda) - I^*_{\mathrm{K1}}\|_2}{\|I^*_{\mathrm{K1}}\|_2}, \qquad H_{20} = \frac{\|I_{\mathrm{K1}}(\lambda) - I^*_{\mathrm{K1}}\|_\infty}{\|I^*_{\mathrm{K1}}\|_\infty}.$$

Here, v_{max} and c_{max} denote the maximum value of the membrane potential and the calcium concentration, respectively. Similarly, v_{rest} and c_{rest} denote the resting membrane potential and calcium concentration, respectively, defined as the values obtained 10 ms before stimulation. Moreover, $\|I\|_2$ and $\|I\|_\infty$ are defined as

$$\|I\|_2 = \sqrt{\sum_n I(t_n)^2},$$
$$\|I\|_\infty = \max_n |I(t_n)|,$$

where n runs over all the time steps of an action potential. The currents I_{Na}, I_{CaL}, I_{Kr}, and I_{K1} are chosen to be included in the cost function because we are especially interested in obtaining realistic behaviors for these currents since these are the currents considered in the drug inversions.

4 Identification of simulated single-channel block using H_{V} andH_{Ca}

In Figure 5 of the paper we showed the value of $H_{V+\mathrm{Ca}}$ for pairwise perturbations of the maximum conductance of four major currents for simulated single channel block of each of the currents. Figures S5 and S6 show corresponding plots for the cost functions H_V and H_{Ca}, respectively. In these figures, we observe that the terms of H_V seem to contain the main part of $H_{V+\mathrm{Ca}}$ observed in Figure 5 of the paper.

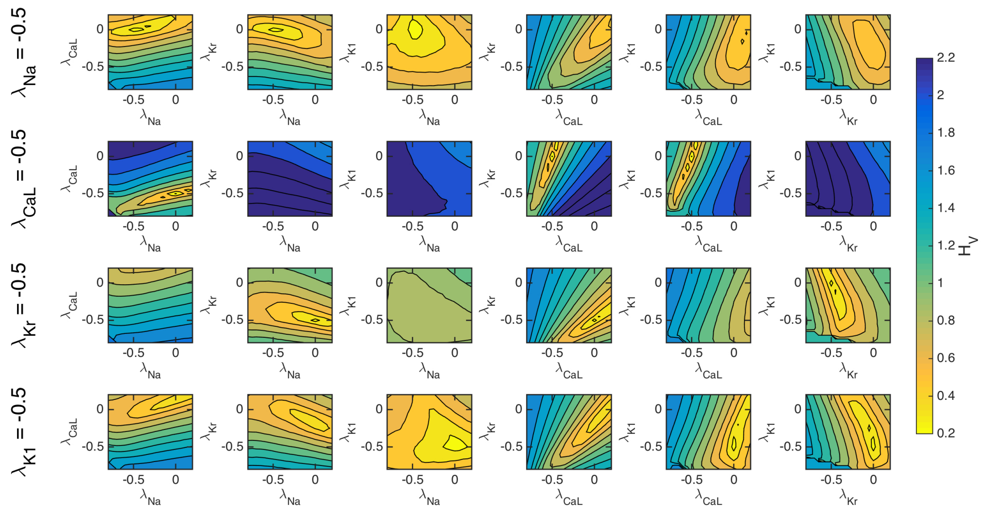

Figure S5: The cost function H_V with $\varepsilon = 0.2$ evaluated for pairwise perturbations of the maximum conductances of four major currents for simulated single-channel block of each of the currents. In the upper panel, I_{Na} is blocked by 50%, and in the next panels, I_{CaL}, I_{Kr} and I_{K1} are similarly blocked by 50%.

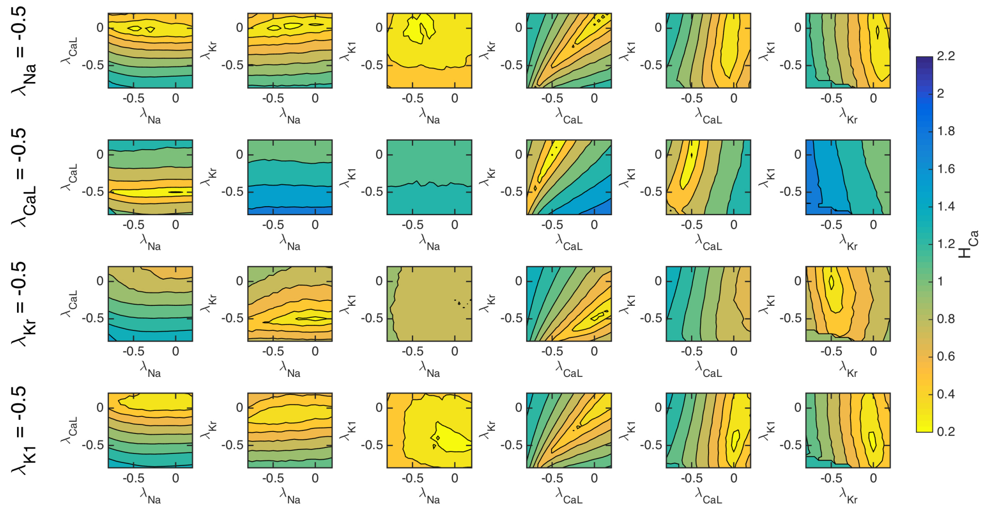

Figure S6: Like Figure S5, except that we cosnider the cost function H_{Ca} instead of H_V.

References

[1] Michelangelo Paci, Jari Hyttinen, Katriina Aalto-Setälä, and Stefano Severi. Computational models of ventricular-and atrial-like human induced pluripotent stem cell derived cardiomyocytes. *Annals of biomedical engineering*, 41(11):2334–2348, 2013.

[2] Anurag Mathur, Peter Loskill, Kaifeng Shao, Nathaniel Huebsch, SoonGweon Hong, Sivan G. Marcus, Natalie Marks, Mohammad Mandegar, Bruce R. Conklin, Luke P. Lee, and Kevin E. Healy. Human ipsc-based cardiac microphysiological system for drug screening applications. *Scientific reports*, 5:8883, 2015.

[3] Thomas J. Hund, Jan P. Kucera, Niels F. Otani, and Yoram Rudy. Ionic charge conservation and long-term steady state in the Luo-Rudy dynamic cell model. *Biophys J*, 81(6):3324–3331, December 2001.

[4] Ronald Wilders. Computer modelling of the sinoatrial node. *Medical and Biological Engineering and Computing*, 45(2):189–207, February 2007.

[5] Victor A. Maltsev, Yael Yaniv, Anna V. Maltsev, Michael D. Stern, and Edward G. Lakatta. Modern perspectives on numerical modeling of cardiac pacemaker cell. *Journal of Pharmacological Sciences*, 125(1):6–38, 2014.

[6] Kirsten H.W.J. ten Tusscher, Denis Noble, Penelope J. Noble, and Alexander V. Panfilov. A model for human ventricular tissue. *American Journal of Physiology-Heart and Circulatory Physiology*, 286(4):H1573–H1589, 2004.
